# The problem with ‘My Five Moments for Hand Hygiene’

**DOI:** 10.1136/bmjqs-2020-011911

**Published:** 2021-07-14

**Authors:** Dinah Gould, Edward Purssell, Annette Jeanes, Nicolas Drey, Jane Chudleigh, Jacob McKnight

**Affiliations:** 1 Independent Consultant, London, UK; 2 School of Health Sciences, City University, London, UK; 3 Infection Medical Chambers Ltd, London, UK; 4 The Health Systems Collaborative, Nuffield Department of Clinical Medicine, Oxford, UK

**Keywords:** infection control, nosocomial infections, patient safety, health policy, quality improvement

*‘The problem with…’ series covers controversial topics related to efforts to improve healthcare quality, including widely recommended, but deceptively difficult strategies for improvement and pervasive problems that seem to resist solution.*

## Introduction

Healthcare-associated infections (HCAIs) are the most common adverse events affecting patients.^1^ The pathogens responsible are often carried on health workers’ hands, and on the evidence of epidemiological and microbiological studies, in theory hand hygiene ought to break the chain of infection.^2^ The WHO^3^ promotes ‘My Five Moments for Hand Hygiene’^4^ as a ‘time-space’ framework to identify points in the sequence of care when hand hygiene should occur to prevent transmission. The Five Moments conceptualise risk in relation to two virtual areas: the patient zone and the healthcare zone.^4^ The patient zone comprises the patient and their immediate surroundings: intact skin and all inanimate surfaces in direct contact with the patient and all the surfaces handled by healthcare workers. The healthcare zone comprises everything outside the patient zone. Except for the original definition of zones provided by Sax *et al*,^4^ no other definition of patient and healthcare zones appears to exist. The model assumes that the healthcare zone is contaminated with potentially harmful micro-organisms (ie, those able to cause exogenous infection and/or resistant to antimicrobials). The Five Moments are the dominant paradigm used to organise practice, policy and research in relation to hand hygiene. In this paper we identify five ‘inconvenient truths’ limiting the Five Moments: (1) the development of the Five Moments did not include the perspectives of stakeholders; (2) it is not always possible to implement Five Moments for all patients all the time; (3) the patient zone is not a fixed entity; (4) the Five Moments overlook barriers that reduce hand hygiene adherence; and (5) adherence to the Five Moments cannot prevent all risks of transmission. These ‘inconvenient truths’ have implications for the way that we conceptualise hand hygiene and measure hand hygiene performance. We propose four solutions to promote hand hygiene. The COVID-19 pandemic has brought rapid change to health services delivery, including all aspects of infection prevention,^5^ and could be the catalyst to update hand hygiene programmes incorporating these solutions.

### The development of the Five Moments did not include the perspectives of stakeholders

The Five Moments predate contemporary guideline development. Newer approaches emphasise the importance of balancing benefits and harms, patient values and preferences, acceptability and equity, as well as feasibility and strength of the evidence in line with the WHO recommendations.^6^ The Five Moments were published over 10 years ago and do not consider the perspectives of health workers, although adherence increases if their views are taken into account.^7^

### It is not always possible to implement the Five Moments for all patients all the time

Accounts of the Five Moments are frequently illustrated with a diagram depicting how pathogens can be transmitted to an acutely sick patient, but patients have widely differing needs and receive care in diverse settings, and the Five Moments do not adapt well to all the many differences between individuals and clinical settings. For example, they do not apply to all inpatients and to only a very small proportion of all the others receiving healthcare. Increasingly patients are older and have chronic conditions, placing them at very high risk of infection, yet receive much or all of their care in outpatient and community settings where delineation into the patient and the healthcare zone is less clear. Many of these patients never occupy the conventional patient zone, or occupy it briefly but might still be at considerable risk, for example those undergoing invasive procedures; pathogens able to cause HCAI have been recovered from health workers’ hands in outpatient clinics with opportunity for transmission.^8^ In low-income countries overcrowding causes physical overlap between neighbouring bedspaces, and the notion that each patient can occupy a discrete zone is untenable.^9^ As it is not always possible to identify which patients are at high risk in a given situation, it is safer to promote hand hygiene throughout healthcare premises regardless of which groups of patients occupy them, for example by providing hand hygiene products at key locations such as ward entry points.

### The patient zone is not a fixed entity

Viewing all hospital premises outside the patient zone as a single, homogenous area oversimplifies the complexity of healthcare environments. First areas with heavy traffic (eg, corridors, foyers) are likely to be more heavily contaminated than wards. Peripatetic health workers and others moving between wards and departments can disseminate large numbers of micro-organisms picked up in general hospital locations, with shedding en route.^10^ Second the patient zone is not a fixed entity. As patients move between clinical areas on the same ward and to non-clinical areas, they carry their microbiota with them. Third microbial shedding contributes to environmental contamination in all the locations in transit and at the destination. Individuals in rooms formerly occupied by infectious patients are at increased risk of colonisation and infection,^11^ although they occupy the same physical space consecutively rather than occupying the same patient zone. Finally many health workers never enter the patient zone, although they handle equipment that are able to operate as fomites that will enter it.

### The Five Moments overlook barriers that can reduce hand hygiene adherence

The Five Moments assume that the decision to undertake hand hygiene is always under the direct control of health workers but they are frequently confronted with competing priorities. Hand hygiene can be compromised by high workload and clinical and non-clinical interruptions.^12^ When the pace of work is rapid, health workers segue between one task and the next without pause, and multitask, particularly in acute care settings^13^. It is not always possible to determine precisely when hand hygiene is necessary.^14^

### Adherence to the Five Moments cannot prevent all risks of transmission

Surfaces distant from patient care areas are often heavily contaminated with pathogens able to cause HCAI. They can withstand desiccation, survive for long periods in the inanimate environment and frequently contaminate health workers’ hands.^15^ If the Five Moments are applied, cross-infection should be avoidable providing hand hygiene is undertaken before the health worker enters the patient zone or initiates contact within it. Unfortunately adherence at these moments is often low,^16–18^ and even if hand hygiene is undertaken it may not be thorough enough to remove all pathogens from the hand surfaces, especially if health workers are busy and hand hygiene episodes are rapid and perfunctory.^19^ Sharing portable equipment (eg, devices to monitor vital signs) between patients and other items (eg, digital technology, pens, clothing) carried into the patient zone present additional risks because decontamination is not feasible with constant movement across zones.^13 20^ Unless health workers conceptualise the patient zone as intended by Sax *et al*,^4^ there may be risk of transmission.^21^ The way that hand hygiene audit is undertaken is known to drift over time within organisations according to local interpretation.^22^ It is likely that health workers’ interpretations of the Five Moments may be subject to drift in the same way.

### Implications for hand hygiene audit

There are challenges to auditing all of the five moments in all health workers who might contribute to contamination and cross-infection in wards. Routine audit is usually restricted to patient care areas, but disposal of body fluids takes place away from the bedside. As a result, data for Moment 3 (after risk of exposure to blood and body fluids) are often missed. Many hand hygiene opportunities exist outside the patient zone, are not encapsulated within the Five Moments and are omitted from hand hygiene audits (eg, after handling potentially contaminated equipment in utility rooms). Health workers not directly attached to wards are frequently excluded. Observation of activities within the patient zone is frequently incomplete because vantage is poor and bedside curtains obscure clinical activities.^23^ Visitors to healthcare facilities are often excluded, although they may contribute to care and their hands may be contaminated by pathogens responsible for HCAI.^24^ Adaptation is necessary before hand hygiene audit tools can be used in settings other than wards, but little guidance is available.

## Proposed solutions

We propose four solutions to contain hand transmission (see table 1) and conclude by considering how the COVID-19 pandemic could help to stimulate change.

**Table 1:** Proposed actions to the inconvenient truths based on the four solutions (proposed solutions in the text)

Inconvenient truths	Proposed actions
Inconvenient truth 1: hand hygiene guidelines need updating.	Rewrite the guidelines for hand hygiene practice and audit based on newer methodologies, for example National Institute for Health and Care Excellence^38^ (solution 1). Consider health workers’ and patients’ preferences and opinions (solution 1).
Inconvenient truth 2: it is not always possible to implement the Five Moments for all patients all the time.	Implement hand hygiene dispensers at hospital, clinics and ward entrances and throughout wards with prompts, monitoring at all locations, publicity and national signage (solution 2).
Inconvenient truth 3: the concept of the patient zone is oversimplified.	Place hand hygiene dispensers at hospital, clinic and ward entrances with prompts, monitoring and publicity (solution 2). Introduce a stochastic approach to hand hygiene programmes and audit at agreed ‘set points’ (solution 3). Refresh hand hygiene training to reflect hand hygiene at the agreed ‘set points’ (solution 3). Introduce non-touch technology (eg, automatic doors) (solution 2).
Inconvenient truth 4: barriers that can reduce hand hygiene adherence are overlooked.	Introduce self-disinfecting surfaces and equipment (solution 4). Increase frequency of cleaning in clinical and non-clinical areas, especially high-contact areas (solution 4). Introduce chlorhexidine gluconate into the formulations of handrubs used in clinical areas (solution 4).
Inconvenient truth 5: adherence to the Five Moments cannot prevent all risks of transmission.	Implement hand hygiene dispensers at hospital, clinic and ward entrances with prompts, monitoring and national signage (solution 2). Introduce a stochastic approach to hand hygiene programmes and audit at agreed ‘set points’ (solution 3). Refresh hand hygiene training to reflect the stochastic approach (solution 3). Introduce self-disinfecting surfaces and equipment. Introduce chlorhexidine gluconate into handrubs used in clinical areas (solution 4). Increase frequency of cleaning in clinical and non-clinical areas, especially high-contact areas (solution 4).

Our first proposed solution is to update the guidelines for hand hygiene to meet contemporary standards, incorporating the opinions and needs of service users and health workers.^6^ Straightforward interventions such as asking about optimal placement of alcohol handrub and positioning dispensers where workflow is high can promote uptake.^25^

Our second proposed solution is to promote rigorous hand hygiene as the norm for everybody in healthcare premises. A national campaign employing the same signage in all locations could be launched in conjunction with non-touch surfaces (eg, automatic door-opening devices). Notices combined with visual or audible alerts can promote uptake at entrances to hospitals,^26^ clinics^27^ and wards.^28^ Consistent use at these locations would prompt hand hygiene at least twice before health workers or visitors reach settings where care is delivered. Devices to promote and monitor uptake at these locations are commercially available.

Our third proposed solution is to enhance hand hygiene at the point of immediate patient care. New hand hygiene programmes based on a stochastic model of transmission could compensate for some of the limitations of the Five Moments. Computer simulations demonstrate that hand contamination and transmission both have random elements.^29^ Individual hand contacts represent low risk of transmission; it is the overall risk at system level and the cumulative frequency of hand contacts that successively increase risk.^30^ Cumulative risk could be overcome by introducing thorough antisepsis at the beginning and end of health workers’ shifts and at predetermined intervals throughout to compensate for hand hygiene opportunities that might be overlooked or inadequately performed. We suggest that these new set points should augment the Five Moments, not replace them. With this system, hand hygiene frequency would require modification according to patient vulnerability and under particular circumstances (eg, if a cluster of infections occurs). Visitors to healthcare facilities would need to be included in these arrangements, especially if they engage in patient care. Audits would need to be adjusted to include adherence at the new set points and to obtain data in relation to both frequency and thoroughness. All those present in the clinical environment throughout an audit period would need to be included. An agile approach adapted in response to frequent adjustments would be required and an audit tool to monitor thoroughness would need to be developed. A new hand hygiene programme based on a stochastic approach could be taken as the catalyst to revitalise clinicians’ hand hygiene. Adjustments to set points and audits to meet immediate clinical need would provide the periodic refreshers that have been identified as necessary to revitalise hand hygiene campaigns.^31^ Health workers will need education and training to promote this new approach to hand hygiene.

Our fourth proposed solution is to reduce the microbial burden of the environment to reduce the risk of microbial transfer into the patient zone. This could be achieved by increasing the frequency of cleaning in clinical and non-clinical areas, especially surfaces that are high-touch, and the use of self-disinfecting surfaces,^32^ equipment^33^ and uniforms.^34^ Chlorhexidine gluconate could be incorporated into alcohol-based hand hygiene preparations used in the patient zone; it has residual bactericidal activity so the effects of antisepsis would be more persistent.^35^

### Capitalising on the COVID-19 pandemic to stimulate change

Although numerous interventions to improve hand hygiene adherence have been reported, sustaining effectiveness is impossible unless campaigns are periodically refreshed.^36^ Successful implementation requires leadership and cooperation throughout the organisation, understanding the context in which care takes place and embedding the intervention into wider patient safety initiatives.^31^ Introducing new hand hygiene interventions fits well alongside the organisation-wide changes introduced to prevent the spread of SARS-CoV-2 and the part played by the infection prevention teams who spearheaded them. Public health messages throughout the pandemic have emphasised the importance of hand hygiene via the media, social media platforms and other advertising outlets.^5^ The challenge is to promote sustainable behaviour change, for example through the national approach suggested in our second solution. Information on health provider websites could emphasise the imperative for everybody to undertake hand hygiene before entering healthcare premises and when moving to different locations within them. Before the COVID-19 pandemic the use of technologies to reduce microbial contamination might have been rejected due to their cost and the lack of belief in the evidence that these technologies are worth the expenditure.^37^ The COVID-19 pandemic has shown the need to invest in infection prevention and the value of employing a range of strategies to reduce risk of transmission. The Five Moments were designed to help health workers identify the points in the sequence of patient care when hand hygiene should occur.^4^ We argue that they now need to be updated to meet contemporary needs in conjunction with other technologies.
